# Fungal Infection Induces Sex-Specific Transcriptional Changes and Alters Sexual Dimorphism in the Dioecious Plant *Silene latifolia*

**DOI:** 10.1371/journal.pgen.1005536

**Published:** 2015-10-08

**Authors:** Niklaus Zemp, Raquel Tavares, Alex Widmer

**Affiliations:** 1 Institute of Integrative Biology (IBZ), ETH Zurich, Zürich, Switzerland; 2 Laboratoire de Biométrie et Biologie Evolutive (UMR 5558), CNRS / Université Lyon 1, Villeurbanne, France; University of California Berkeley, UNITED STATES

## Abstract

Sexual dimorphism, including differences in morphology, behavior and physiology between females and males, is widespread in animals and plants and is shaped by gene expression differences between the sexes. Such expression differences may also underlie sex-specific responses of hosts to pathogen infections, most notably when pathogens induce partial sex reversal in infected hosts. The genetic changes associated with sex-specific responses to pathogen infections on the one hand, and sexual dimorphism on the other hand, remain poorly understood. The dioecious White Campion (*Silene latifolia*) displays sexual dimorphism in floral traits and infection with the smut fungus *Micobrotryum lychnidis-dioicae* induces a partial sex reversal in females. We find strong sex-specific responses to pathogen infection and reduced sexual dimorphism in infected *S*. *latifolia*. This provides a direct link between pathogen-mediated changes in sex-biased gene expression and altered sexual dimorphism in the host. Expression changes following infection affected mainly genes with male-biased expression in healthy plants. In females, these genes were up-regulated, leading to a masculinization of the transcriptome. In contrast, infection in males was associated with down-regulation of these genes, leading to a demasculinization of the transcriptome. To a lesser extent, genes with female-biased expression in healthy plants were also affected in opposite directions in the two sexes. These genes were overall down-regulated in females and up-regulated in males, causing, respectively, a defeminization in infected females and a feminization of the transcriptome in infected males. Our results reveal strong sex-specific responses to pathogen infection in a dioecious plant and provide a link between pathogen-induced changes in sex-biased gene expression and sexual dimorphism.

## Author Summary

Females and males differ from each other in many traits, including morphology, behavior and physiology. Differences in gene expression between the sexes, known as sex-biased gene expression, contribute to such sexual dimorphism. Here we characterize the responses of females and males of the dioecious plant *Silene latifolia* to infection with the anther smut fungus *Micobrotryum lychnidis-dioicae*. This fungus sterilizes the plant and induces a partial sex reversal in female hosts that form rudimentary stamens, thus allowing the fungus to transmit its spores via pollinators. Our comparisons of gene expression in healthy and infected plants reveal strong sex-specific responses to anther smut infection. Expression changes in females and males are in opposite directions and are associated with reduced sexual dimorphism between infected females and males. Our study reveals that infection with the anther smut fungus alters the extent of sex-biased gene expression in *S*. *latifolia* in a sex-specific manner and highlights how transcriptomic changes in females and males shape sexual dimorphism.

## Introduction

Sexual dimorphism is widespread in animal and plant species with separate sexes although females and males are often genetically very similar. Genes that are predominantly expressed in one sex over the other, also known as genes with sex-biased expression, may be involved in some of these differences [1,2]. Genes with sex-biased expression often accumulate on sex chromosomes and contribute importantly to the expression of sexually dimorphic traits [1,3], as for instance in *Drosophila melanogaster* [4] or mice [5]. In mammals a substantial proportion of genes also show sex-biased expression in somatic tissues, including liver [5] and brain [6], where they contribute to complex physiological and behavioral differences between the two sexes. Host responses to parasite and pathogen infections may also be sex-specific and lead to different effects on female and male hosts [7,8]. Sex-specific transcriptional changes upon pathogen infection, however, remain poorly understood [9,10] and have not been reported to date in plants.

Parasites may have strong effects upon their hosts, for example on morphology, ecology, life history or behavior, but the underlying mechanisms are often unknown [11,12]. One of the most remarkable host modifications upon infection is sex reversal. *Wolbachia* bacteria can induce nearly complete feminization of their arthropod hosts [13] and some amphipod crustacean species are feminized by the parasite *Nosema granulosis* [14]. These sex reversals can be associated with changes in sexually dimorphic traits. However, despite the strong interest in the genetic basis of sex determination and sexual dimorphism on the one hand, and the genetics of host-parasite interactions on the other hand, the molecular changes associated with the induced morphological alterations in the host have not been elucidated in animals and plants. In plants, sex reversals upon pathogen infection are rare in species with separate sexes, but evidence for partial sex reversal exists for dioecious *Silene* species that are infected by the sterilizing anther smut fungus *Microbotryum [15].*

*Silene latifolia* Poiret is a dioecious plant with heteromorphic sex chromosomes that has become a model system for investigating the evolution of separate sexes and sex chromosomes [16]. The sex chromosomes of *S*. *latifolia* are evolutionarily young but show many characteristics that are also known from much older animal sex chromosome systems [17], such as Y chromosome degeneration [18–21] and evidence for dosage compensation [19] but see [22–24]. Moreover, *S*. *latifolia* females and males display strong sexual dimorphism in reproductive and vegetative traits [25].

Smut fungi of the genus *Microbotryum* infect many members of the genus *Silene* [26]. The life cycle of the anther smut *Micobrotryum lychnidis-dioicae* is strictly associated with its host plant *S*. *latifolia*. Fungal teliospores are produced in the host’s anthers and are transmitted by insect vectors to healthy hosts. Upon infection, the host plant is sterilized. Infection of female plants in dioecious hosts could potentially be a dead end for the fungus, because stamen development is suppressed in healthy females. Yet, infection with *M*. *lychnidis-dioicae* induces the formation of rudimentary stamens in infected females [27], thus leading to a partial sex reversal in the host. Consequently, rudimentary stamens that aid spore dispersal are formed in both host sexes upon smut infection.

The *S*. *latifolia*–*M*. *lychnidis-dioicae* system has been widely studied as a model for host-pathogen co-evolution [28–30] and has been used for studies on the genetic control of sexual differences in *S*. *latifolia*. The gene *SLM2*, for example, has been reported to be differentially expressed between infected and control females and it has been suggested that this gene may be involved in stamen formation [31]. More extensive studies of transcriptional changes in this host–parasite system were hindered by the complexity and size (1C ~2.5 Gb) of the largely unexplored host genome and the difficulties associated with studying expression changes in large numbers of genes simultaneously.

Most studies performed on the *S*. *latifolia*–*M*. *lychnidis-dioicae* system have used Northern blotting and *in situ* hybridization to assess gene expression differences between the sexes or between healthy and infected plants [31–33]. Recent advances in next-generation sequencing (NGS) methods now allow investigating host and parasite gene expression patterns simultaneously across both genomes, for example in a dual RNA-seq approach [34]. Thereby transcriptomes of both infected and healthy hosts are sequenced and compared to investigate expression changes simultaneously in the host, the parasite and their interaction *in situ*. Importantly, this approach is also feasible in species with hitherto largely uncharacterized and complex genomes and may therefore expand the range of host-parasite systems that can be analyzed at the molecular level.

In this study, we used an RNA-seq approach to investigate how the *S*. *latifolia* transcriptome changes upon infection by *M*. *lychnidis-dioicae*, which induces a partial sex reversal in female host plants. We found that smut fungus infection led to major changes in the host transcriptome and that these expression changes were highly sex-specific. The transcriptional changes reduced the extent of sex-biased expression between infected female and male *S*. *latifolia* plants and were associated with a partial sex reversal in females and reduced sexual dimorphism in floral traits, providing a direct link between sex-biased gene expression and phenotypic differences between the sexes.

## Results

### RNA-seq statistics

For the expression analysis we obtained 6.4 billion Illumina paired-end reads across the twelve sequenced libraries, corresponding to 64 Gigabases (Gb) of RNA-seq data that are publicly available (BioProject ID: PRJNA285435). On average, 89.67% of the reads were mapped to the *S*. *latifolia* reference transcriptome [19] (S1 Table). Putative sex-linked contigs were inferred in a previous study through segregation analysis [19] and have alleles that are expressed from the X and Y chromosome. After discarding contigs with low read numbers, we retained 86,824 non sex-linked and 1,564 putative sex-linked contigs that were used for further analyses. The 'non sex-linked' contigs encompass primarily autosomal contigs but may also include pseudoautosomal and sex-linked contigs that were not identified as sex-linked in the original segregation analysis [19] because of low expression or lack of sex-linked SNPs in the cross used.

### Gene expression changes in *S*. *latifolia* upon *M*. *lychnidis-dioicae* infection

Our analysis revealed that gene expression changes upon infection of female and male *S*. *latifolia* with *M*. *lychnidis-dioicae* are not only dependent on infection *per se* but also on host sex. Infection led to expression changes in 4.7% of the non sex-linked and 7.2% of the sex-linked contigs only, whereas 9.8% of the non sex-linked and 16.0% of the sex-linked contigs displayed sex-dependent differences in gene expression (Table 1). The interaction effect between sex and treatment was stronger than the main treatment effect, with 5.0% of the non sex-linked and 9.6% of the sex-linked contigs having a significant interaction effect (Table 1). As a consequence, we further analyzed gene expression changes upon infection separately for *S*. *latifolia* females and males. We also assessed whether contigs differ in expression levels between the sexes and thus showed sex-bias in expression. Contigs without detectable expression in one sex were here called sex-limited in expression. We further tested whether contigs with sex-biased expression responded more strongly to infection in females and males than contigs without sex-bias (i.e. unbiased) in expression.

**Table 1:** Effects of infection, host sex and their interaction on gene expression in *S*. *latifolia*. Numbers of non sex-linked (86, 824) and sex-linked (1, 564) contigs (and percentages) that are differentially expressed as a consequence of infection with *M*. *lychnidis-dioicae*, host sex and the interaction between infection and host sex. The model with two factors (infection and sex) and their interaction was calculated.

	Infection		Sex		Infection X Sex	
contig	#	%	#	%	#	%
non sex-linked	4045	4.7	8488	9.8	4300	5.0
sex-linked	113	7.2	251	16.0	150	9.6

### Sex-specific effects of *Microbotryum* infection on gene expression in flower buds of female and male *S*. *latifolia*

We found significant changes in gene expression upon *M*. *lychnidis-dioicae* infection in 3.7% of non sex-linked and 8.1% of sex-linked contigs in *S*. *latifolia* females. In males, 4.7% of non sex-linked and 7.2% of sex-linked contigs showed altered expression upon infection. Significantly higher proportions of sex-linked than non sex-linked contigs responded to infection in both females and males (χ^2^-tests, P-values ≤ 0.001; Table 2). Further, proportions of contigs with altered expression in infected plants were significantly higher for contigs with sex-biased expression (63.6% of the non sex-linked and 77.7% of the sex-linked contigs) than for contigs with unbiased expression in healthy plants (2.3% of the non sex-linked and 2.9% of the sex-linked contigs; χ^2^-tests, P-values ≤ 0.001; S2 Table).

**Table 2:** Gene expression changes upon infection in female and male *S*. *latifolia*. Numbers and percentages of differentially expressed contigs in infected females and males for non sex-linked (86, 824) and sex-linked (1, 564) contigs only. Significantly more changes were found in sex-linked than in non sex-linked contigs (χ^2^-tests tests, P-values ≤0.001).

	non sex-linked		sex-linked	
plant sex	#	%	#	%
females	3233	3.7	127	8.1
males	4045	4.7	113	7.2

**Fig 1:**
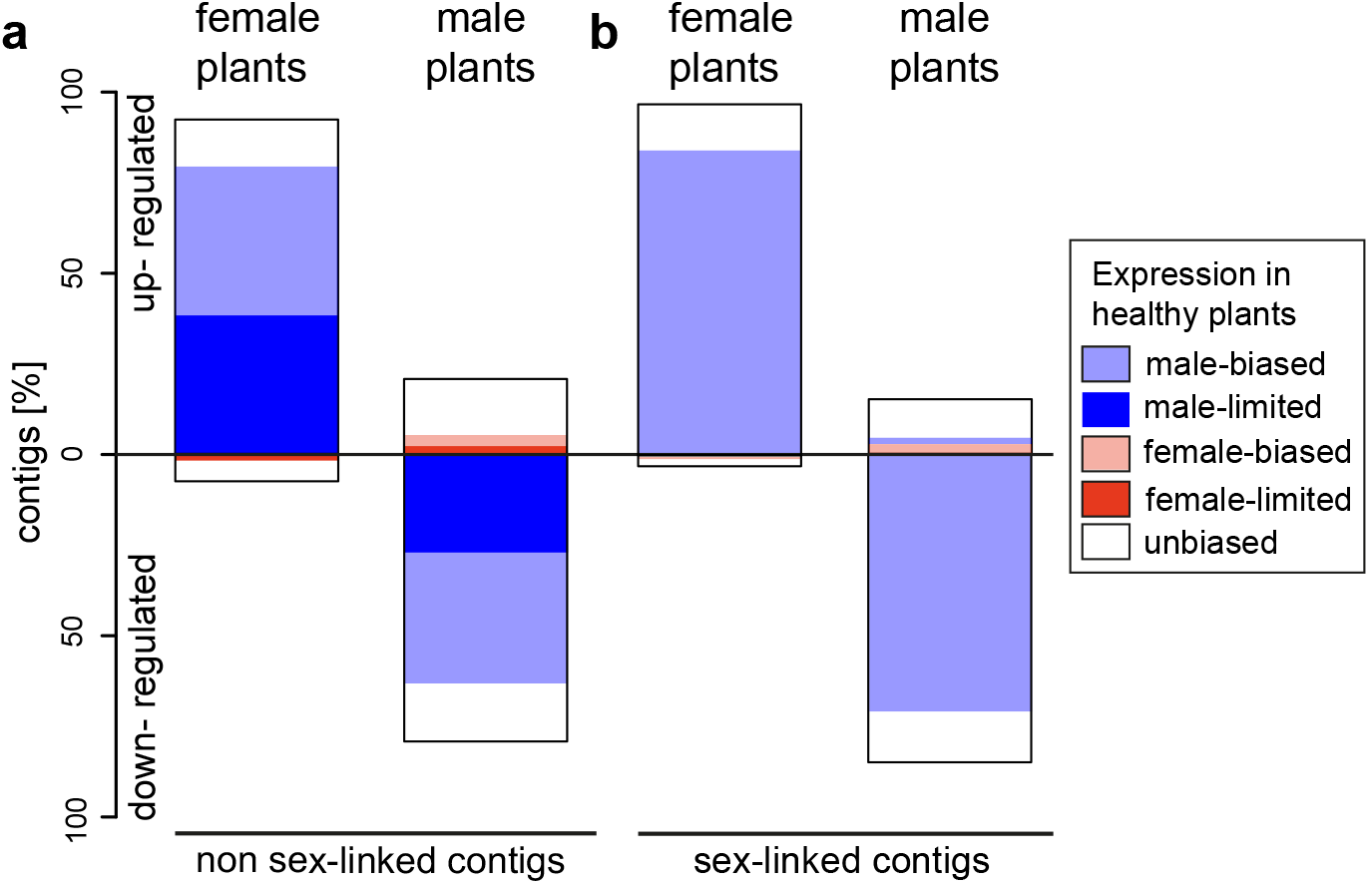
Gene expression changes following infection with *M*. *lychnidis-dioicae* in female and male plants of *S*. *latifolia* for non sex-linked and sex-linked contigs. Bars indicate proportions of significantly up-regulated or down-regulated contigs in infected plants relative to healthy plants for (a) non sex-linked and (b) sex-linked contigs only. Inferences about sex linkage were taken from a previous study [19]. Proportions of contigs with female-biased expression in healthy plants are in pink, proportions of contigs with male-biased expression in light blue and proportions of contigs with unbiased expression are in white. Dark red areas represent proportions of contigs with female-limited expression (exclusively expressed in healthy females). Dark blue areas represent contigs with male-limited expression (exclusively expressed in healthy males) (for exact values see S3 Table and S4 Table).

**Fig 2:**
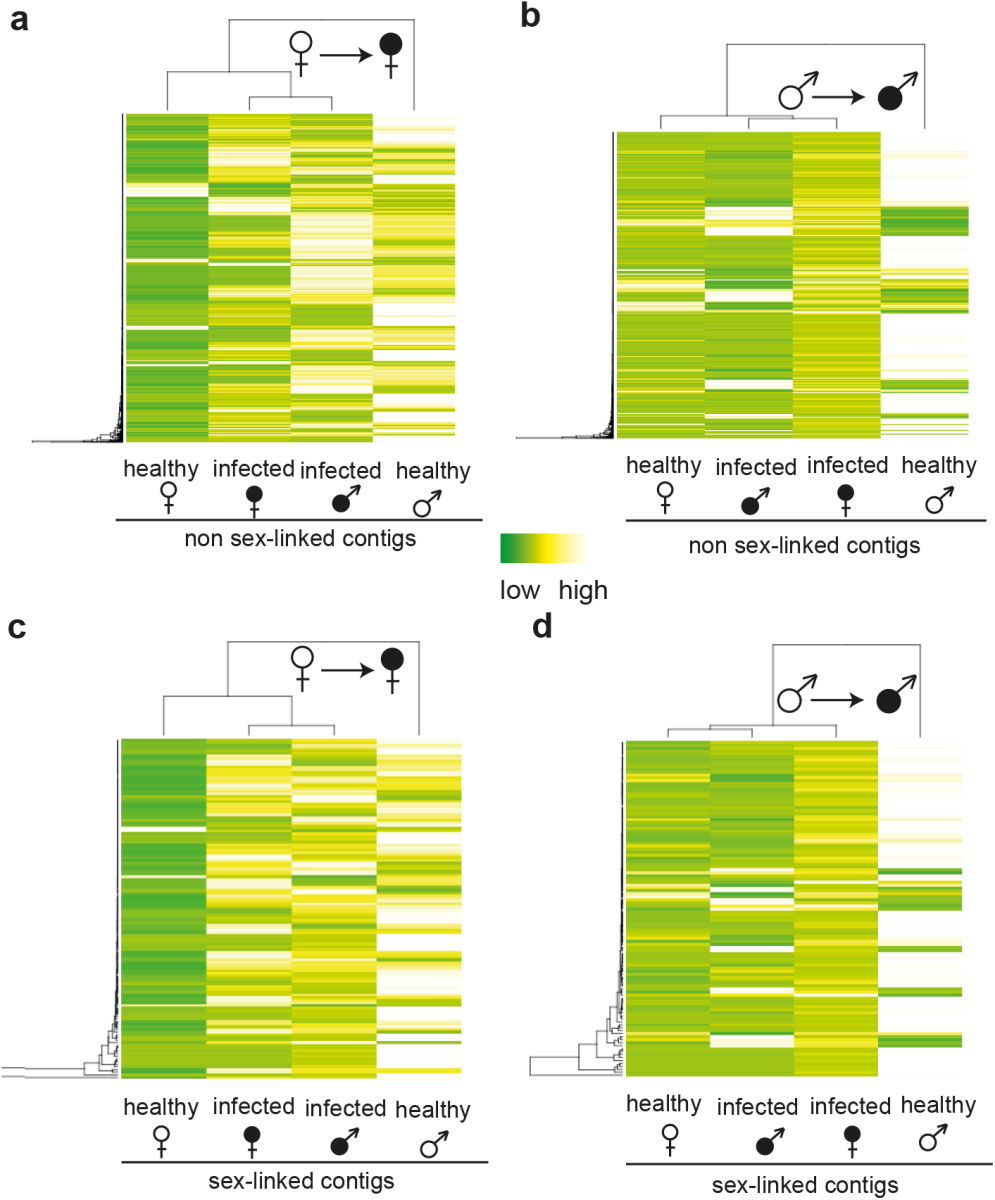
Heat maps and clustering of gene expression in non-infected and infected female and male *S*. *latifolia*. Heat maps and hierarchical clustering analyses of expression patterns for contigs that are significantly differentially expressed between healthy and infected females (a, c) and males (b, d) for non sex-linked (a, b) and sex-linked (c, d) contigs. Dark green colour indicates low expression and white high gene expression intensity.

In infected females, 92.4% and 96.9% of expression changes in non sex-linked and sex-linked contigs were due to increased expression relative to healthy females (Fig 1). A strong positive correlation was observed between the extent of male-biased expression in healthy plants and expression change upon infection for both sex-linked and non sex-linked contigs (non sex-linked: ρ = 0.53, sex-linked: ρ = 0.58; S1 Fig). Most up-regulated contigs in infected females were originally categorized as male-biased in healthy plants (non sex-linked contigs: 86%, sex-linked contigs: 87%). The remaining up-regulated contigs in infected females displayed no sex-bias in expression in healthy plants (Fig 1, S3 Table). In contrast, none of the contigs with female-biased expression in healthy plants were up-regulated upon infection in females. A small proportion of all contigs that responded to infection in females were down-regulated (non sex-linked: 7.6%, sex-linked: 3.1%). Of these, the great majority had no sex-bias in expression in healthy plants, but 19.2% and 25% of down-regulated non sex-linked and sex-linked contigs, respectively, had female-biased expression in healthy plants (Fig 1, S3 Table). Heat maps and clustering of contigs that were differentially expressed upon infection revealed that expression patterns of infected females were more similar to infected males than to healthy females (Fig 2).

In contrast to females, expression changes in males involved mostly a reduction of expression in infected plants relative to healthy ones (79.2% and 85% of non sex-linked and sex-linked contigs, respectively; Fig 1). A strong negative correlation between the extent of male-biased expression in healthy plants and expression change upon infection in males was observed (non sex-linked contigs: ρ = -0.36, sex-linked contigs: ρ = -0.32) (S1 Fig) and most down-regulated contigs displayed male-biased expression in healthy plants (non sex-linked contigs: 78.3%, sex-linked contigs: 84.4%). Increased expression upon infection was found for 20.8% and 15% of non sex-linked and sex-linked contigs, respectively. About two thirds of these contigs displayed no sex-biased expression in healthy plants (Fig 1, S3 Table). Heat maps and clustering of contigs that were differentially expressed between infected and healthy males grouped infected males most closely with healthy females (Fig 2).

### Activation and inactivation of genes with sex-limited expression in healthy plants

We found in both host sexes that several contigs with sex-limited expression (i.e. contigs that are exclusively expressed in one sex) in healthy plants were activated upon infection in the opposite sex. 48.5% of all male-biased contigs that were up-regulated upon infection in female plants were not expressed in healthy females (Fig 1). Of the contigs that were categorized as female-biased in healthy plants and up-regulated upon infection in males, 42.0% had female-limited expression in healthy plants (for absolute values see S4 Table).

Several contigs with sex-limited expression in healthy plants were down-regulated upon infection in the same sex. 10.6% of contigs that were down-regulated upon infection in females were female-limited in expression in healthy plants. In males, 43.4% of contigs that were down-regulated upon infection had male-limited expression in healthy plants, and 61% of these contigs were no longer expressed upon infection in males (for absolute values see S4 Table).

### GO term enrichment analysis

The percentage of annotated contigs per category varied and ranged from 18.75% to 34.14% for non sex-linked and from 47.06% to 75.0% for sex-linked contigs. The majority of over and under-represented GO terms of the differentially expressed genes after infection were shared between females and males (S5 Table), but genes involved in reproductive processes were over-represented among genes that were up-regulated upon infection in females. In males, genes involved in biotic stimulus and defense response were over-represented among up-regulated genes (S5 Table). No GO term enrichment analysis could be performed for non sex-linked and sex-linked, down-regulated contigs in females and for sex-linked, up-regulated contigs in males because of the low numbers of contigs in these categories (S3 Table).

### Changes in sexual dimorphism and sex-biased expression

**Fig 3:**
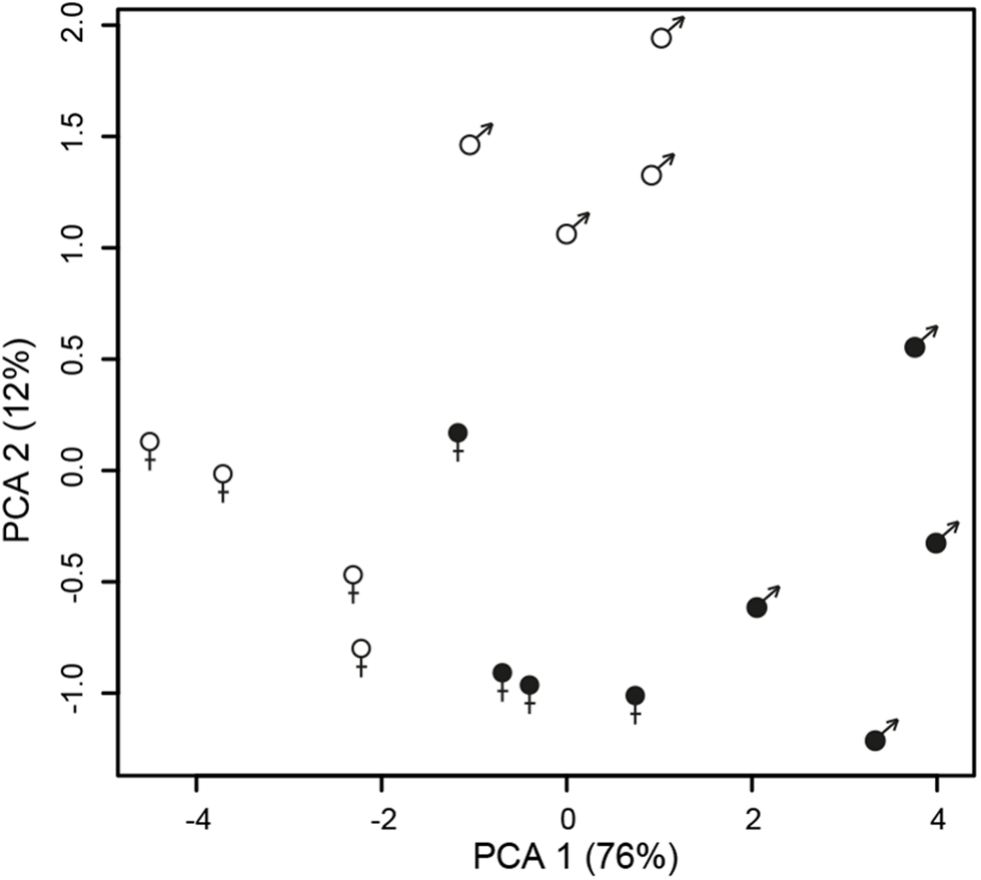
Changes in sexual dimorphism in floral traits between infected (filled symbols) and healthy (open symbols) plants. Principle component analysis (PCA) of floral traits in healthy and infected females and males of *S*. *latifolia*.

**Fig 4:**
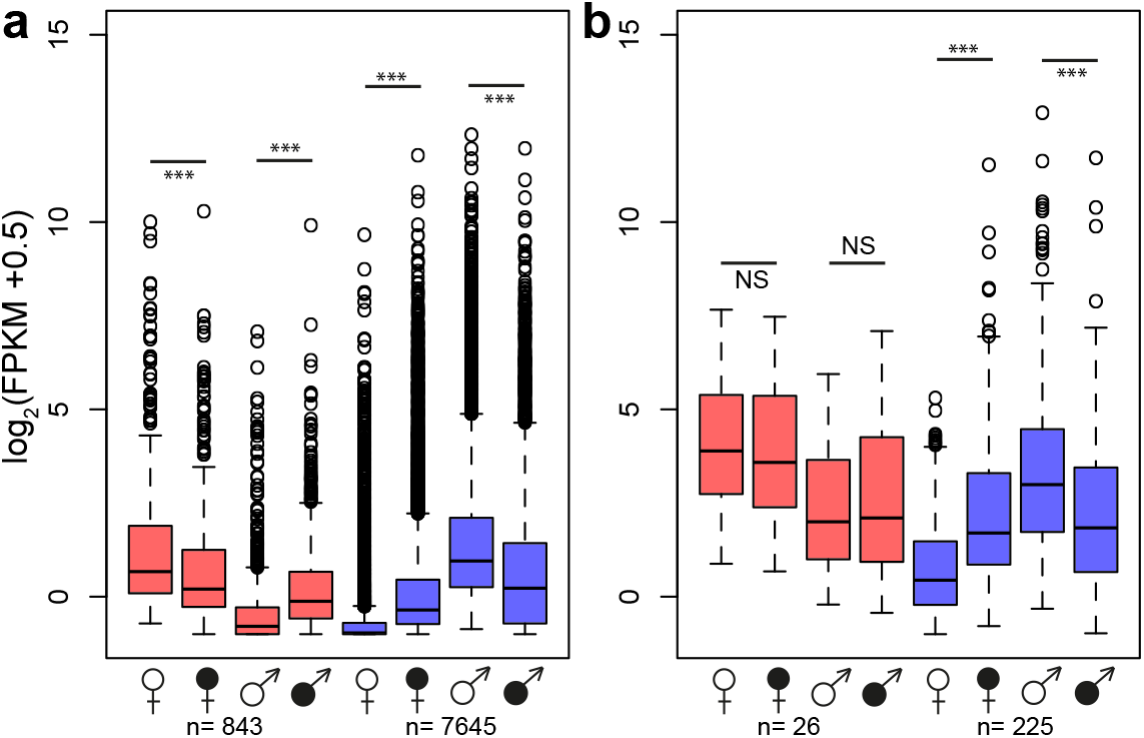
Changes in gene expression intensity of genes with sex-biased expression in healthy *S*. *latifolia* following *Microbotryum* infection. Boxplots show log_2_ expression intensities in FPKM of healthy (open symbols) and infected (filled symbols) plants for contigs with female-biased (red) and male-biased (blue) expression in healthy plants for non sex-linked (a) and the sex-linked (b) contigs only. Asterisks indicate significant differences using Wilcoxon-tests (***: P-value ≤ 0.001; NS: P-value>0.5). Numbers of contigs with sex-biased expression in healthy plants are shown below. Infection induced defeminization (e.g. reduced expression of genes with female-biased expression) and masculinization (e.g. increased expression of genes with male-biased expression) in females and feminization and demasculinization in males.

A principle component analysis (PCA) based on secondary sexually dimorphic traits showed a clear separation between healthy female and male *S*. *latifolia* but reduced sexual dimorphism between infected females and males (Fig 3). Infection led to a shift of females along PCA 1 and in males along both PCA 1 and PCA 2. All measured traits contributed to these changes. At the transcriptome level, sex-bias was significantly reduced upon infection in the plants (χ^2^-tests, P-values ≤ 0.001; S6 Table). 5177 (60.5%) non sex-linked and 172 (68.5%) sex-linked contigs with sex-biased expression in healthy plants no longer exhibited sex-biased expression in infected plants. The analysis of genes that responded significantly to infection and had sex-biased expression in healthy plants (Fig 1) revealed that infection led to defeminization (e.g. down-regulation of genes with female-biased expression) and masculinization (e.g. up-regulation of genes with male-biased expression) in infected females, relative to healthy females, and to feminization and demasculinization in infected males (Fig 4). Expression intensities between healthy and infected plants were significantly different for non sex-linked contigs with female-biased and male-biased expression (Wilcoxon-tests, P-value ≤ 0.0001) and for sex-linked contigs only in contigs with male-biased expression in healthy plants (Wilcoxon-tests, contigs with male-biased expression in both sexes: P-values ≤ 0.001, female-biased infected plants: P-values > 0.5) (Fig 4).

## Discussion

### Sex-specific transcriptional changes in hosts with separate sexes

Parasite infections can have substantial effects on hosts and in species with separate sexes, these effects may differ between host females and males [8]. We found that in the *S*. *latifolia—M*. *lychnidis-dioicae* host-pathogen system, smut infection had strong effects on host gene expression, but sex-specific effects and the interaction effect between host sex and smut infection were even stronger (Table 1, Fig 1). Such sex-specific gene expression changes following pathogen infection have–to the best of our knowledge—not previously been reported in plants, but are known from a limited number of studies in animals, including humans [35] and mice [10]. It is thus crucial to analyze transcriptional changes caused by pathogen infections separately in female and male hosts.

### Effects of *Microbotryum* infection on gene expression in *S*. *latifolia*

Pathogen infections in plants typically induce a complex gene expression response in their hosts [36,37]. 4.7% of all non sex-linked contigs and 7.2% of the sex-linked contigs revealed significant expression differences between infected and healthy *S*. *latifolia* plants (Table 2). In comparison to other analyses of transcriptional changes in plant-pathogen interactions, these values are relatively high. For instance, gene expression responses to nematode infections encompassed 3–4% of genes in *Arabidopsis thaliana* [38] and 0.2–2.3% of genes in rice [39]. The large numbers of differentially expressed genes upon smut infection suggests that infections caused complex expression changes in the host, the extent of which was not revealed by earlier studies [31–33].

Defense related genes are often up-regulated in hosts in response to pathogen infection [40]. Contrary to expectations, GO terms related to defense response (as well as response to biotic stimulus) were over-represented only among genes up-regulated in infected *S*. *latifolia* males (S5 Table), but not in infected females. *S*. *latifolia* males often start to flower earlier and produce more flowers than females, which might increase their risk of infection ([41], but see [42]), and also increases their attractiveness to pollinators [43,44]. Female flowers, on the other hand, have a longer life-span, which may also increase their long-term risk of infection, relative to the short-lived male flowers [30,45]. Whether the difference observed between the sexes of diseased plants also corresponds to the immediate response to pathogen arrival is currently an open question. Further, responses to different stimuli (extracellular, biotic and abiotic) and stresses may also be involved in host responses to parasites. In this case, evidence for a regulatory response to pathogen infection was observed in both sexes. In general, the GO annotation of the *S*. *latifolia* transcriptome should be interpreted with caution. GO annotations are mostly based on the model organism *A*. *thaliana*. The lineages leading to *A*. *thaliana* on the one hand and *S*. *latifolia* on the other hand have diverged approximately 180 million years ago (MYA) [46] and predictions of molecular and biological functions of genes in *S*. *latifolia* from results obtained for *A*. *thaliana* may be inaccurate ([47]). Furthermore, defense related genes are often rapidly evolving [48] and many such genes may therefore not be annotated in *S*. *latifolia*, leading to an underestimation of defense-related expression response. Indeed, only a small proportion of *S*. *latifolia* contigs, ranging from 26.63% for non sex-linked to 57.30% of sex-linked contigs, were annotated and GO terms are often shared between females and males. These potential limitations notwithstanding, *S*. *latifolia* females and males do seem to respond differently upon *M*. *lychnidis-dioicae* infection with respect to the activation of annotated defense-related genes.

### Sex-specific responses to infection

Parasite induced differences between the sexes are well documented in animals [8,49,50] and have also been reported in plants [51–53], where much less is known about sex-specific transcriptomic responses to infection. The few animal studies that have been published indicate that effects on gene expression patterns can be large [35,54], even though these studies did not include alterations of host sexual phenotype. In the dioecious *S*. *latifolia*, infection led to a partial phenotypic sex change in female host plants, but not in males, and sexual dimorphism in floral traits was reduced in infected plants. In a similar way sex-specific responses were observed at the transcriptomic level and reveal that the infection is causing sex-specific transcriptional responses.

Our study revealed that the expression of a disproportionately large number of genes with sex-biased and sex-limited expression in healthy plants changed upon infection and these expression changes led to a substantial change in the ‘transcriptome dimorphism’ between the sexes of infected plants. In females, the great majority of expression changes following infection involved up-regulation of genes that displayed male-biased expression in healthy plants (Fig 1). In marked contrast, expression changes in males largely involved down-regulation of genes with male-biased expression in healthy plants. Interestingly, we found opposite correlations between the extent of sex-biased expression in healthy plants and the sign of expression change upon infection in females and males (S1 Fig). In females, a strong positive association was found, indicating that the more genes displayed male-biased expression in healthy plants, the stronger they were up-regulated in infected females (S1 Fig). Nevertheless, expression intensities rarely reached those observed in healthy males (Fig 2). In contrast, in males, a negative correlation was found between the extent of sex-biased expression in healthy plants and expression change upon infection, indicating that the most strongly male-biased genes in healthy plants were the most strongly down-regulated upon infection in males (S1 Fig). As a consequence, expression levels in infected males of many otherwise male-biased genes in healthy males reached the low expression levels typically observed in healthy females, and many genes were turned off or down-regulated upon infection (S4 Table, numbers in brackets). Thus, expression changes following infection rendered infected females more male-like and *vice versa* for infected males (see also Fig 4).

Masculinization and defeminization of the transcriptome in infected females is in line with their partial sex reversal which allows the fungal pathogen to successfully complete its life cycle for successful transmission [27]. Interestingly, we observed the activation of genes that are not normally expressed in healthy females, i.e. genes with male-limited expression. These may be required for the development of rudimentary stamens and are activated upon infection. This assumption is supported by our finding of an over-representation of genes involved in reproductive processes among up-regulated genes in infected females. The expression of otherwise male-specific genes in infected females indicates that these genes are not located on the Y chromosome and suggests that their suppression in healthy females is under the control of one or several master regulatory genes involved in the genetic control of sex determination. Such regulatory genes may also affect the expression of male-biased genes in healthy plants and contribute to their up-regulation in infected females.

In infected males we found opposite patterns of demasculinization and feminization of the transcriptome (Fig 4). Many genes with male-biased expression in healthy plants were down-regulated following infection (Fig 1). This finding is in line with the observation that sex determination in plants is often regulated by general pathways, such as the ethylene pathway in melon [55]. Such pathways typically have many other cellular functions in addition to sex determination. It is therefore reasonable to expect that at least some of the physiological and transcriptomic differences between females and males are caused by pleiotropic effects of sex determining genes. Expression of genes with male-related function, such as pollen development, is not necessary in infected plants, as fungal spores will replace pollen grains in the anthers. If the expression of such genes is costly, their down-regulation may help reduce energetic costs in the host, so as to be reallocated into other host traits that increase the reproductive success of the fungus [56].

### Sex chromosome effects

The proportion of significantly differentially expressed genes between infected females and males was affected by sex-linkage; the number and proportion of differentially expressed sex-linked genes was higher in infected females than in infected males (Table 2). Morphologically, infected females develop rudimentary stamens from which fungal spores are released [27]. It is tempting to speculate that the development of otherwise male-specific tissues is associated with the higher proportion of differentially expressed sex-linked genes in infected females. Both, theoretical and empirical studies indicate that genes with sex-biased expression preferentially accumulate on the sex chromosomes and may be important for the expression of sexual dimorphism [1,3], as observed for example by the accumulation of sexually dimorphic traits on the *D*. *melanogaster* sex chromosomes [4]. In *S*. *latifolia*, quantitative trait loci (QTL) underlying some sexually dimorphic flower traits (numbers of flowers, calyx width and petal limb length) were found to map to the sex chromosomes [25]. These and similar traits also differ between healthy and infected females and males in our study (S6 Table). Our results thus are in line with the expectation that genes on the *S*. *latifolia* sex chromosomes contribute to the formation of sexually dimorphic and sex-specific structures, but future work needs to be done to quantify sex chromosome effects and identify causal genes.

### Fungus-mediated changes in host morphology

A recent study in birds [57] reported a striking association between the degree of sex-biased gene expression and sexual dimorphism. Smut infection in *S*. *latifolia* leads to a congruent result. The strong transcriptomic changes and the reduced sex-bias in gene expression following smut infection are reflected in the reduced sexual dimorphism observed in infected plants. It has long been noted that infected females become more similar to males through the development of rudimentary stamens [27]. Our morphological measurements further indicated that other sexually dimorphic traits are also altered, as observed in other studies [16], and morphological differences between the sexes are reduced in infected *S*. *latifolia*. This observation is in agreement with reports of more similar flower numbers in infected plants compared to healthy plants [53] but effects of *Microbotryum* infections on alterations of host traits depend on plant and fungal genetic background and are highly variable [52,58]. Some of the changes in host phenotype may be adaptive for the parasite, such as the fungus-induced shift from vegetative growth to flowering, which may increase pathogen reproductive success [51,52], whereas other phenotypic changes induced in the host may be side effects of the infection. Overall, the strong transcriptomic changes in opposite directions induced by the pathogen in female and male hosts observed in this study lead to complex changes in host morphology and phenology, and the partial sex reversal induced in female plants ensures pathogen transmission also from female hosts. Nevertheless, spore production per flower remains greater for male hosts than for females [52,58] indicating that the partial sex change induced by *M*. *lychnidis-dioicae* cannot fully compensate for the lack of Y-linked genes promoting male function in the host.

### Conclusions

We found that infection of the dioecious plant *S*. *latifolia* with the fungal pathogen *M*. *lychnidis-dioicae* led to substantial and highly sex-specific transcriptomic changes in the host. In particular, the expression of genes with male-biased expression in healthy plants was altered upon infection. In female hosts, most gene expression changes involved up-regulations, whereas down-regulations dominated expression changes in infected males. The strong response of sex-linked genes to pathogen infection further suggests that genes on the sex chromosomes are important for the development of male sexual organs and their activation contributes to the defeminization and masculinization of infected *S*. *latifolia* females. In contrast, we found evidence for specific down-regulation upon infection of genes with male-biased expression in healthy males, which was not expected based on the observed morphological changes, and may help to reallocate resources to aid fungal reproduction.

## Material and Methods

### Plant and fungal growth and infection

Haploid strains A1 and A2 of *Micobrotryum lychnidis-dioicae* isolated from *S*. *latifolia* were obtained from M. Hood (University of Virginia, Department of Biology, Charlottesville, USA) and were grown on Potato Dextrose Agar. *S*. *latifolia* seeds from an inbred line that has been propagated by brother-sister mating for 11 generations were used in the present study. Seeds were sterilized in a solution containing 2% bleach (10%), 20% Ethanol (100%) and 0.2% Triton (100%) and were then transferred to water agar. After germination, seedlings in the infection group were inoculated twice with 10^7^ cells each of strains A1 and A2 in sterilized water, whereas the control group was treated twice with sterilized water only. One week later, seedlings were transplanted to pots (13 cm in diameter) filled with Biouniversalerde (Oekohum GmbH, Switzerland). All plants were grown in a greenhouse at Eschikon (Switzerland) with 16 h of light, and temperatures of 22°C during the day and 18°C at night.

### RNA isolation and library construction

Eight small flower buds (5–6 mm; corresponding to stage 11 as defined in [59]) were collected from three infected females and three infected males and three healthy females and three healthy males upon initiation of flowering as described in Zemp et al. [60]. We had three biological replicas per treatment and sex. High quality RNA was extracted as described in Zemp et al. [60] twice independently from buds without calyxes of each infected and healthy plant and the two separate extractions per plant were then pooled. 12 individually tagged libraries (one per sample) were produced with the Illumina TruSeq RNA Preparation Kit v2 with a median insertion size of 200 bp. Libraries were sequenced in three channels on an Illumina HiSeq 2000 at the D-BSSE (ETH Zürich, Switzerland) using 100 bp paired-end reads.

### Read mapping, normalization and quantification of expression differences

Plant RNA-seq reads were mapped against the *S*. *latifolia* reference transcriptome [19] with BWA (v. 0.5.9-r16) [61] allowing up to five mismatches per read. Putative sex-linked contigs were inferred in a previous study based on a segregation analysis [19]. Repetitive contigs [62] were masked using RepeatMasker [63] and we retained only contigs that had at least ten mapped reads per library. To jointly assess the effects of the *M*. *lychnidis-dioicae* infection and plant sex on gene expression changes we first used a statistical model that takes both factors, infection and plant sex, as well as their interaction into account using edgeR based on a common dispersal and the default normalization method [64]. We identified the numbers of differentially expressed contigs with a FDR ≤0.05 for each factor. Because of the strong interaction between infection and plant sex (Table 1) we simplified the model and analyzed the two sexes independently using contrasts in edgeR. We identified significantly differentially expressed contigs between infected females and males (referring to contigs with sex-biased expression in infected plants), healthy females and males (referring to contigs with sex-biased expression in healthy plants), healthy and infected females, and healthy and infected males. We further assessed the proportions of contigs with sex-limited expression (i.e. exclusively expressed in one sex) in healthy and infected plants for the non sex-linked contigs. For the sex-linked contigs, this was not possible, because the identification of sex-linkage was based on the assumption that X and Y alleles are expressed [19]. Several comparisons were performed with χ^2^-tests in R [65]. Mean expression values in FPKM (fragments per kilobase of transcript per million fragments) were calculated for the infected and the healthy females and males. Spearman correlations between the extent of sex-bias in healthy plants and the extent of up-regulation in infected plants were computed using R. Heat maps and hierarchical cluster analysis were performed for the differentially expressed contigs upon infection in females and males using the function heat maps in R. Differences in expression intensities for contigs with female- and male-biased expression in healthy plants were tested between infected and healthy plants using Wilcoxon tests.

### Gene Ontology (GO) and enrichment analysis

All contigs were annotated using the Blast2go [66] pipeline and enrichment tests upon infection were performed for the non sex-linked contigs with an FDR ≤0.05. Because of the small number of sex-linked contigs, it was not possible to use FDR and we therefore used a p ≤ 0.05 significance threshold.

### Morphological measurements

Morphological measurements on traits, which were known to be dimorphic [25], were performed on four individuals per sex and treatment. In each individual we measured plant height at peak flowering and counted three times the numbers of flowers/buds produced per side branch. Pictures of fresh and dissected flowers were taken with a digital camera (Coolpix P7700, Nikon) and the following traits were measured using ImageJ [67] flower diameter, calyx length and width, tooth length and petal blade and stalk length of five flowers (S2 Fig). Trait means were calculated for each of the five flower traits per individual and principle component analysis (PCA) was performed using R.

## Supporting Information

S1 Fig Expression changes in contigs with sex-biased expression upon *Microbotryum* infection in female and male *S*. *latifolia*. Log_2_ fold change of expression in healthy males over females (i.e. extent of sex-bias expression) was plotted against log_2_ fold change of expression in infected over healthy females (a, b) and males (c,d), for non sex-linked (a,c) and sex-linked contigs (b,d). Numbers in graphs are ρ values of Spearman correlation coefficients.
(TIF)

S2 Fig Morphological traits measured in *S*. *latifolia* flowers. Five floral traits were measured, including flower diameter, calyx length, calyx width, calyx tooth length, and petal blade and stalk length. In addition, plant height and numbers of flowers/buds per side branch were measured.
(TIF)

S1 Table Samples. Overview of RNA-seq libraries used in the present study with information on the number of nucleotides sequenced per library (in Gb), nucleotides mapped and percentage of reads mapped.
(XLSX)

S2 Table Effects of infection on genes with sex-biased and unbiased expression in healthy plants. Numbers and proportions of non sex-linked and sex-linked contigs that are differentially expressed upon infection and display sex-biased and unbiased expression, respectively, in healthy plants. A significantly higher proportion of contigs with sex-biased expression was differentially expressed upon infection than contigs with unbiased expression (χ^2^-tests, P-values ≤ 0.001).
(XLSX)

S3 Table Categorisation of differentially expressed contigs in infected plants. Absolute numbers and proportions of differentially expressed non sex-linked and sex-linked contigs in *S*. *latifolia* females and males (Fig 1). Bold lines indicate the numbers and proportions of contigs that are up-regulated or down-regulated upon infection. Below are numbers and proportions of contigs with female-biased, male-biased, and unbiased expression in the healthy plants.
(XLSX)

S4 Table Gene expression changes upon infection for genes with sex-biased expression in *S*. *latifolia* females and males. Absolute values and proportions of sex-biased contigs that are differentially expressed in infected plants are given for female-biased and male-biased genes (Fig 1). Female-limited and male-limited genes are subsets of female-biased and male-biased genes, respectively, and are characterized by their expression in one sex only. Values in brackets are numbers of contigs for which no expression was detected in infected plants.
(XLSX)

S5 Table Results of GO enrichment analysis for non sex-linked and sex-linked contigs. Significantly over and under-represented GO terms for non sex-linked (FDR ≤ 0.05) and sex-linked (p<0.05) contigs that are up-regulated in infected females, down-regulated in infected males, and up-regulated in infected males.
(XLSX)

S6 Table Morphological and transcriptional differences between infected and healthy *S*. *latifolia* females and males. Mean trait values and standard errors (SE) for each trait measured in infected and healthy females and males (n = 4) are given. Numbers and proportions of non sex-linked and sex-linked contigs with sex-biased expression are indicated, as well as numbers and proportions of contigs with female- and male-biased expression in healthy and infected plants.
(XLSX)
