# Correction: Fungal Infection Induces Sex-Specific Transcriptional Changes and Alters Sexual Dimorphism in the Dioecious Plant *Silene latifolia*

**DOI:** 10.1371/journal.pgen.1005662

**Published:** 2015-11-17

**Authors:** The PLOS Genetics Staff

The editor listed on this article is incorrect. The correct editor is: Rodney Mauricio, University of Georgia, United States. The publisher apologises for the error.
