# Supplementary figures and images for: Fungal Infection Induces Sex-Specific Transcriptional Changes and Alters Sexual Dimorphism in the Dioecious Plant Silene latifolia
Source: PLoS Genet. 2015 Oct 8;11(10):e1005536. doi: 10.1371/journal.pgen.1005536 (PMC4598173; doi:10.1371/journal.pgen.1005536)

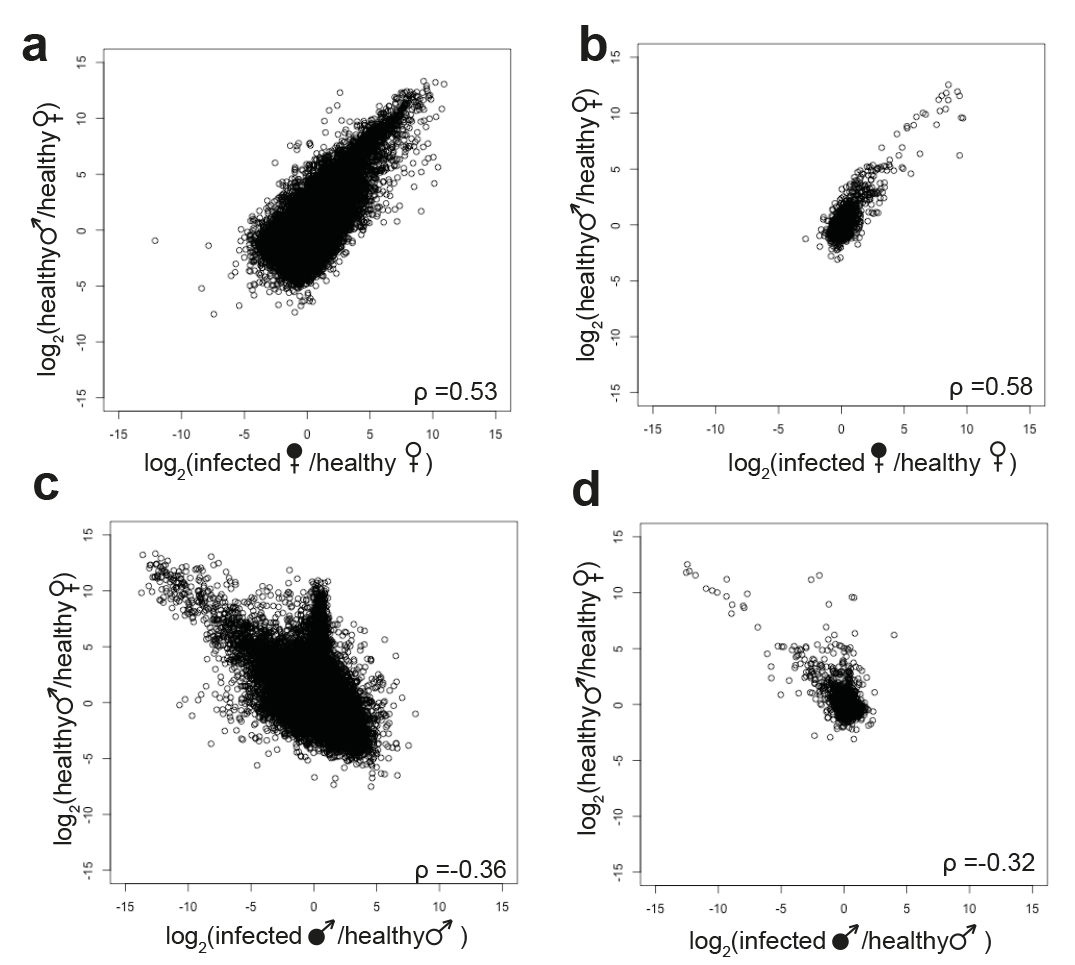

Supplement: S1 Fig — Log2 fold change of expression in healthy males over females (i.e. extent of sex-bias expression) was plotted against log2 fold change of expression in infected over healthy females (a, b) and males (c,d), for non sex-linked (a,c) and sex-linked contigs (b,d). Numbers in graphs are ρ values of Spearman correlation coefficients. (TIF) [file pgen.1005536.s001.tif]

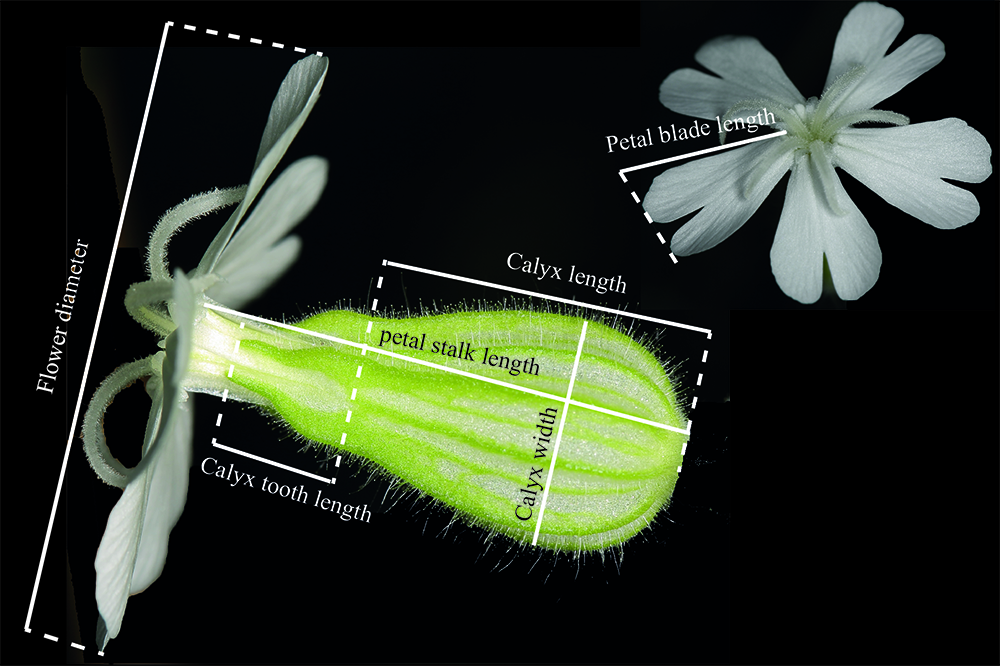

Supplement: S2 Fig — Five floral traits were measured, including flower diameter, calyx length, calyx width, calyx tooth length, and petal blade and stalk length. In addition, plant height and numbers of flowers/buds per side branch were measured. (TIF) [file pgen.1005536.s002.tif]
